# Genetic variants associated with the occurrence and progression of adolescent idiopathic scoliosis: a systematic review protocol

**DOI:** 10.1186/s13643-022-01991-8

**Published:** 2022-06-09

**Authors:** Elizabeth A. Terhune, Patricia C. Heyn, Christi R. Piper, Nancy Hadley-Miller

**Affiliations:** 1 Department of Orthopedics, University of Colorado Anschutz Medical Campus, Aurora, CO USA; 2 Department of Physical Medicine and Rehabilitation, University of Colorado Anschutz Medical Campus, Aurora, CO USA; 3 Center for Gait and Movement Analysis, Children’s Hospital Colorado, Aurora, CO USA; 4 Cochrane US University of Colorado Anschutz Medical Campus, Aurora, CO USA; 5 Strauss Health Sciences Library, University of Colorado Anschutz Medical Campus, Aurora, CO USA; 6 Musculoskeletal Research Center, Children’s Hospital Colorado, Aurora, CO USA

**Keywords:** Adolescent idiopathic scoliosis (AIS), Genetic studies, Genome-wide association, Exome sequencing, Whole genome sequencing, Targeted sequencing, Systematic review, Variants, Protocol

## Abstract

**Background:**

Adolescent idiopathic scoliosis (AIS) is a structural lateral spinal curvature of ≥ 10° with rotation. Approximately 2–3% of children in most populations are affected with AIS, and this condition is responsible for approximately $1.1 billion in surgical costs to the US healthcare system. Although a genetic factor for AIS has been demonstrated for decades, with multiple potentially contributory loci identified across populations, treatment options have remained limited to bracing and surgery.

**Methods:**

The databases MEDLINE (via PubMed), Embase, Google Scholar, and Ovid MEDLINE will be searched and limited to articles in English. We will conduct title and abstract, full-text, and data extraction screening through Covidence, followed by data transfer to a custom REDCap database. Quality assessment will be confirmed by multiple reviewers. Studies containing variant-level data (i.e., GWAS, exome sequencing) for AIS subjects and controls will be considered. Outcomes of interest will include presence/absence of AIS, scoliosis curve severity, scoliosis curve progression, and presence/absence of nucleotide-level variants. Analyses will include odds ratios and relative risk assessments, and subgroup analysis (i.e., males vs. females, age groups) may be applied. Quality assessment tools will include GRADE and Q-Genie for genetic studies.

**Discussion:**

In this systematic review, we seek to evaluate the quality of genetic evidence for AIS to better inform research efforts, to ultimately improve the quality of patient care and diagnosis.

**Systematic review registration:**

PROSPERO registration #CRD42021243253

**Supplementary Information:**

The online version contains supplementary material available at 10.1186/s13643-022-01991-8.

## Introduction/background

**Fig. 1:**
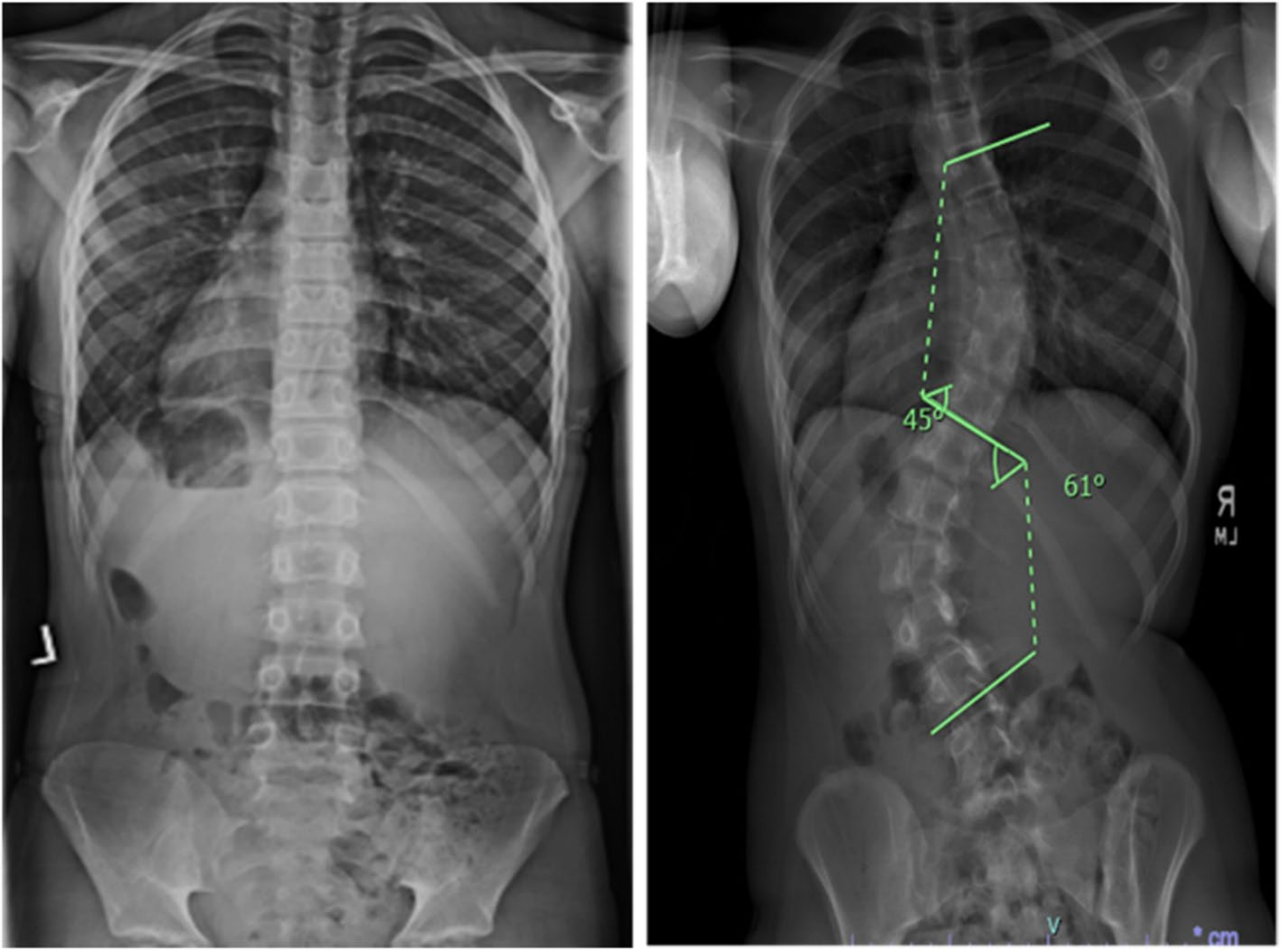
Radiographs of children with a normal spine (left) and severe adolescent idiopathic scoliosis (AIS) (right), shown with Cobb angle measurements

Adolescent idiopathic scoliosis (AIS) is the most common pediatric spinal deformity, affecting 2–3% of otherwise healthy children, with a 9:1 ratio of affected females:males for severe curvatures [1, 2]. AIS is defined as a structural lateral spinal curvature of ≥10° and typically manifests during the pre-adolescent period of rapid growth velocity [3–5]. Radiographs of adolescents with a normal spine and with AIS, as measured by Cobb angle, are provided in Fig. 1. The high prevalence of AIS across populations, combined with potential morbidities related to functional deformity, social stigma, back pain, surgical interventions, and disease, have prompted costly school screening programs for early detection of scoliosis. The true clinical dilemma is to determine which children are at risk for AIS, and once diagnosed, which children are at risk for significant curve progression. Treatment options for scoliosis have remained stagnant for decades, and spinal fusion surgery is often advised for severe progressive curvatures with life-long implications. Together, the annual public health cost of pediatric screening, specialty referrals, bracing and surgery for AIS exceeds $3 billion USD annually, not accounting for adult morbidities, including chronic back pain, pulmonary and neurological complications, and secondary surgeries, thus contributing to the estimated $849 billion in annual costs for musculoskeletal conditions [6–9].

Decades of research into AIS families and population studies have established the strong heritability of AIS with significant sibling recurrence-risk ratio for both mild and severe curvatures [10, 11]. While genetic heritability is high, studies of AIS families and large cohorts have consistently demonstrated significant heterogeneity indicative of the complex genetic nature of this disorder [12, 13]. Whole exome sequencing (WES) and genome-wide association studies (GWAS) have resulted in a large number of potential predisposition genetic variants, such as those in or near *LBX1*, *BNC2*, and *GPR126*, which have been replicated across multiple populations. However, these variants correspond to only a modest increase in risk for AIS. Additionally, the biological roles of the most promising genetic variants in relation to AIS are unknown; thus, they have not led to a mechanistic understanding of the pathology related to this disorder.

The use of genetic variants in the clinical realm as therapeutic targets has achieved some clinical utility, particularly in relation to breast and colon cancer, and, more recently, with the potential modulation of viruses such as COVID-19 [14, 15]. In relation to AIS, in 2010, a genetic screen of 53 single nucleotide polymorphisms (SNPs) for Caucasian children ages 9–13 years with mild scoliosis was designed to predict those most at-risk of severe curve progression [16]. The genetic screen marketed as the ScoliScore (Transgenomics, Inc.) garnered mixed results upon validation, and it is unclear whether it offers any information for clinical decision-making beyond a patient’s natural history [17–20]. Nevertheless, genetic research related to AIS has continued to expand exponentially, with large population-based genome-wide association studies (GWAS) [21–27] and whole-exome sequencing (WES) [28–36] studies in both families and populations. These studies vary widely based on population, methodology, number of subjects and controls, statistical analyses, and interpretation of results [5, 37–40]. A critical analysis of the existing information would be of great importance, not only to assist in research efforts related to AIS genetics, but also to aid in our ability to identify children for the onset of AIS, to prioritize those children at risk for AIS progression, and to develop targeted therapeutic interventions for a personalized medicine approach to this disorder [41]. This manuscript proposes a systematic review to assist in this effort.

Our primary objective within this systematic review is to identify specific risk single-nucleotide polymorphisms (SNPs) for AIS through methodologies that provide significant variant level data (i.e., GWAS, next-generation DNA sequencing) on the diagnosis of AIS and, if diagnosed, the susceptibility to AIS curve progression.

### Aims and objectives

Our overarching aim is to inform clinicians and researchers of the current state of adolescent idiopathic scoliosis (AIS) genetic data to better inform future research efforts, with the ultimate goal of helping to discover clinically actionable targets. We plan to follow a critical appraisal approach to accomplish each of the following aims:

1. To evaluate, summarize, and synthesize literature on the genetics of AIS, in order to provide recommendations for further genetic studies or further functional work, with emphasis on the development of potential diagnostics and prognostics;
2. To summarize overall study information (i.e., publication country of origin, ethnicities of study populations, area of PI expertise) from the current body of literature of AIS genetics; and
3. To determine the quality of current literature on AIS genetics, by GRADE [42–44] and Q-Genie [45] evaluation, including the level of evidence.

By doing this work, we expect to inform the field of the most well-replicated and robust genetic variants linked to AIS risk to date. We expect to summarize overall study information to provide a snapshot of the field of AIS genetics, and in the process, we expect to identify high-priority research areas where we have identified significant gaps in the present literature. We plan to identify the pros and cons of specific methodologies and provide guidance for future studies. Lastly, we expect to provide relevant information for future prioritization of basic studies as well as translational research, in the hopes of developing future diagnostics and prognostics.

## Methods/design

**Fig. 2:**
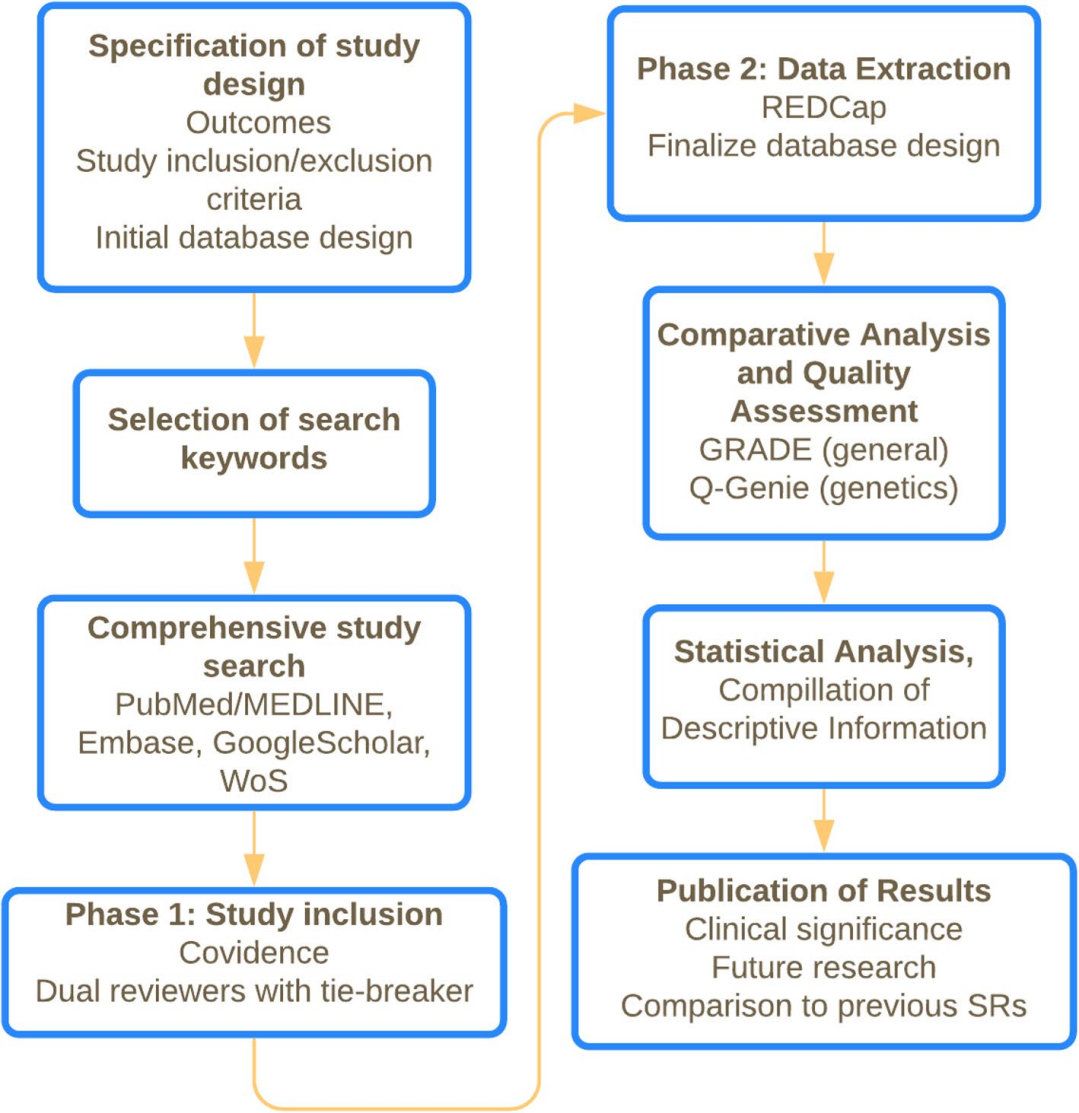
Flow chart overview of study design

This systematic review protocol was designed in accordance with the Preferred Reporting Items of Systematic Reviews and Meta-Analysis for Protocols (PRISMA-P) v2015 checklist (Supplemental File 1). The research group will follow current best practices from Cochrane guidance and recommendations for systematic reviews [46]. We will follow the standards for best practices for transparent, reproducible, and ethical reporting of systematic review guided by the PRISMA-P statement [47–49]. An overview of the methodology for this systematic review is provided in Fig. 2.

### Eligibility criteria

The protocol has been registered in PROSPERO [50] (CRD42021243253). Included studies must focus on AIS and genetic testing. Studies to be included must report data at the nucleotide level, such as genome-wide association studies (GWAS), targeted sequencing, whole genome sequencing, and whole exome sequencing. Candidate gene studies will be eligible, but must be a validation of a previous whole genome study (i.e., whole exome sequencing, GWAS). Studies will be excluded if they report linkage or other data not at the nucleotide level, report transcriptomic and proteomic data, or contain incomplete datasets. Additionally, case-only studies or studies containing fewer than 100 subjects in the case cohort will be excluded. Conference abstracts, editorials, and reviews will also be excluded. Included studies will be limited to English language publications.

### Information sources

A comprehensive literature search will be designed and performed by a medical librarian (CP) for the concepts of AIS and genetic testing. Relevant publications will be identified by searching the following databases with a combination of standardized index terms and keywords: Ovid MEDLINE ALL (1946 to present), Embase (via Elsevier, 1947 to present), MEDLINE (via Pubmed.gov), Web of Science Core Collection (via Thomson Reuters, including Science Citation Index Expanded 1974 to present, and Social Sciences Citation Index 1974 to present), and Google Scholar (where Publish or Perish software [51] allowed downloading of the first 200 search results). Searches will be limited to English language studies. The preliminary search strategies were conducted in April 2021 and identified 1297 records. The search strategies were peer-reviewed by an external librarian using the Peer Review of Electronic Search Strategies checklist [52]. All results will be exported to and deduplicated in EndNote 20 (Clarivate). Covidence systematic review software (Veritas Health Innovation) will be used for screening and full-text review. See Supplemental File 2 for a list of all database search strategies.

### Study selection

Citations and abstracts will be uploaded into Covidence for study selection*.* The study selection process is organized into two levels. For level one screening, two authors (ET, PH, and/or NHM) will independently screen all titles and abstracts. Data will be compiled at which time consensus will be reached by discussion on any disagreements for exclusion. For level two screening, full-text articles considered for inclusion will be independently reviewed by two authors. Consensus will be reached by discussion on any disagreements for inclusion. Studies will be excluded if the study cohort is an infantile population.

### Inclusion and exclusion criteria

Full inclusion and exclusion criteria are provided in Supplemental File 3. In brief, the inclusion criteria will encompass genetic studies investigating specific variants (i.e., GWAS, targeted sequencing, whole genome sequencing, whole exome sequencing). Exclusion criteria includes linkage studies and other genetic studies that do not provide variant-level resolution. Candidate studies that are not validating a previous whole-genome study will also be excluded. Review articles, articles not available in English, studies without control populations, studies with case cohorts <100 subjects, systematic reviews, and meta-analyses will also be excluded. Lastly, articles published before 2011 will be excluded.

### Data extraction and synthesis

The research group will first create an Excel spreadsheet to collect relevant information from the genetic studies, followed by the creation of a REDCap database (securely managed by the University of Colorado) after the most relevant study metrics have been determined. Key data for extraction will include, but will not be limited to, the number of case and control individuals, study inclusion and exclusion criteria, basic clinical and demographic information of cases and controls, genetic methodology used, statistical methods used, significant genetic variants identified and associated *p* values, the number of individuals in case and control groups with risk variants identified in the study. The study team will select the first round of studies to be extracted with the aid of the medical librarian. Team members will review and approve all studies to be included and excluded in the SR and will ensure that all methods are transparent, unbiased, and complete. Study team will meet weekly during the duration of the study.

### Quality assessment

#### GRADE

The proposed review will use the Grading of Recommendations Assessment, Development and Evaluation (GRADE) guidelines to determine the quality and strength of recommendations [42–44]. Quality will be adjudicated as high (further research is very unlikely to change our confidence in the estimate of effect), moderate (further research is likely to have an important impact on our confidence in the estimate of effect and may change the estimate), low (further research is very likely to have an important impact on our confidence in the estimate of effect and is likely to change the estimate), or very low (very uncertain about the estimate of effect).

#### Q-Genie

We will also evaluate genetic association study quality using Q-Genie, an evaluation tool to rank genetic studies on 11 criteria based on previous study recommendations  [45]. This tool includes ranking studies as “low,” “moderate,” or “high” quality and assists in the selection of studies for inclusion. Risk-of-bias for individual studies will be assessed using both GRADE and Q-Genie.

### Study outcomes

Main study outcomes will include presence/absence of AIS, scoliosis curve severity, scoliosis curve progression, and presence/absence of nucleotide-level variants. Analyses will include odds ratios and relative risk assessments, and subgroup analysis (i.e., males vs. females, age groups) may be applied.

## Expected results

We expect a significant degree of heterogeneity across the genetic and statistical methodologies used, sample and control groups, thresholds of significance, results, and interpretation of results across studies. We expect several findings, particularly those that have been replicated across multiple studies and populations, to be regarded as high quality by our evaluation.

We expect the results generated by GWAS and next-generation or targeted sequencing methodologies to produce largely different results. GWAS tends to find SNPs that are common within the general population (have a high minor allele frequency), whereas next-generation sequencing methodologies may apply bioinformatic filters that contain only rare or uncommon variants [53]. Additionally, some next-generation sequencing methodologies only look at particular areas of the genome (i.e., exome sequencing analyzes coding regions only). Thus, we expect different sequencing methodologies to produce a different collection of causal variants for AIS.

We also expect a high degree of overall genetic heterogeneity for AIS, with the possibility of specific SNPs showing association with specific ethnic populations or subtypes of AIS [38]. Overall, we expect studies to support a significant genetic contribution to AIS etiology [5, 38, 39, 54–60].

Lastly, we expect certain study populations to be well-represented in the literature of the genetics of AIS, while we expect other ethnic groups (i.e., populations of African descent) to be understudied. Identifying understudied populations and other gaps in the current literature will assist us in making recommendations for areas of high-priority research. We anticipate that the outcomes of this systemic review will indicate that several variants implicated by GWAS and replicated across multiple ethnic groups, including *LBX1* [22, 23, 26, 27, 31, 61–73] and *GPR126* [24, 27, 73–78], will statistically associate with an increased risk of AIS development. Furthermore, we anticipate that rare or uncommon variants within extracellular matrix genes [29, 32, 34, 79–81] will collectively increase the risk of AIS. Once the systematic review is complete, results will be disseminated through both scientific, peer-reviewed journal article(s) and national conference presentations. Any amendments made to this protocol when conducting the study will be amended in PROSPERO and reported in the final manuscript.

## Discussion

This review builds upon current literature to critically assess genetic variants associated with AIS risk. A recent systematic review, using eight studies from 1950 to 2017 that met inclusion criteria, found moderate evidence that did not clarify a single-gene basis of AIS [82]. The mixed results of their study led the authors to recommend that AIS researchers consider etiological factors beyond genetics alone. Three of the eight included studies supported a single-gene hypothesis for AIS etiology, albeit within specific populations. Additional systematic reviews of AIS have evaluated the quality of evidence between AIS etiology and specific variants, including those in or near *LBX1* [64–66, 69, 72, 83], *ESR1/2* [84–86], and *VDR* [87, 88]. Our systematic review will build upon this work by evaluating evidence for specific SNPs in relationship to AIS etiology, rather than collective evidence for a single-gene hypothesis. Based on our preliminary search strategies, we also expect to evaluate a much larger pool of research articles (>500 articles vs. 36 articles [82]).

### Potential challenges

We anticipate several potential challenges with this study. First, as we are including several sequencing methodologies in this study, we anticipate a lack of common data elements across studies that may prove challenging for extraction. Second, we anticipate that some included studies will have missing data. AIS is a common disorder and, without proper verification by radiograph or physical exam, individuals with mild scoliosis can be mistakenly counted as controls.

### Potential limitations

Potential limitations of this study include a lack of thorough reporting within studies (for example, inclusion/exclusion criteria, bioinformatic and statistical analyses, control databases used, lack of appropriate matching of cases and controls). A second limitation is an underrepresentation of non-Caucasian study subjects within sample populations. We also anticipate a potential lack of methodological rigor in included studies, particularly inappropriate biostatistical methodologies or inappropriate sample populations.

### Study implications

This study will inform best practices for future genetic studies of AIS and help researchers to prioritize specific genetic loci that may warrant further research. Additionally, this study will provide a foundation for the creation of clinical genetic diagnostics to help inform a child’s risk of AIS development or severe curvature progression, a matter of great importance in pediatric orthopedics.

## Supplementary Information

**Additional file 1.** PRISMA

**Additional file 2.** Search Strategies

**Additional file 3.** Inclusion Exclusion

## Data Availability

The data used for this study will be extracted from publications within the search databases, including PubMed, GoogleScholar, and Ovid Medline, as outlined in the Methods section.

## Abbreviations

AIS: Adolescent idiopathic scoliosis
GWAS: Genome-wide association study
WES: Whole exome sequencing
WGS: Whole genome sequencing
