# Supplementary material for: Genetic variants associated with the occurrence and progression of adolescent idiopathic scoliosis: a systematic review protocol
Source: Syst Rev. 2022 Jun 9;11:118. doi: 10.1186/s13643-022-01991-8 (PMC9178937; doi:10.1186/s13643-022-01991-8)
Supplement: Supplementary file 2 — Additional file 2. Search Strategies [file 13643_2022_1991_MOESM2_ESM.docx]

Supplemental Material: Search Strategies

|  | MEDLINE via PubMed.gov |
| --- | --- |
| Query # | Search Strategy |
| #1 | Whole Genome Sequencing[Mesh] OR Genetic Association Studies[Mesh] OR Genome-Wide Association Study[Mesh] OR DNA Copy Number Variations[Mesh] OR Sequence Analysis, DNA[Mesh] OR Sequence Analysis, RNA[Mesh] OR High-Throughput Nucleotide Sequencing[Mesh:NoExp] OR LBX1 protein, human[Supplementary Concept] OR ADGRG6 protein, human[Supplementary Concept] OR TBX6 protein, human[Supplementary Concept] OR Genetic Testing[Mesh] OR Genotyping Techniques[Mesh] OR Polymorphism, Single Nucleotide[Mesh] OR "Genetic Predisposition to Disease"[Mesh] OR Sequence Analysis[Mesh] OR genetic stud*[tw] OR genetic variant*[tw] OR isolated candidate gene stud*[tw] OR DNA sequenc*[tw] OR RNA sequenc*[tw] OR next generation sequenc*[tw] OR copy number variat*[tw] OR LBX1[tw] OR GPR126[tw] OR TBX6[tw] OR Single Nucleotide Polymorphism*[tw] OR Copy Number Polymorphism*[tw] OR Copy Number Change*[tw] OR Genetic Association Stud*[tw] OR Genotype-Phenotype Association*[tw] OR Genotype Phenotype Correlation*[tw] OR Candidate Gene Analys*[tw] OR Candidate Gene Identification*[tw] OR Gene Discover*[tw] OR Candidate Gene Association Stud*[tw] OR Complete Genome Sequenc*[tw] OR Whole Genome Sequenc*[tw] OR Whole Transcriptome Sequenc*[tw] OR Complete Transcriptome Sequenc*[tw] OR Whole Exome Sequenc*[tw] OR Complete Exome Sequenc*[tw] OR Complete Exome Sequenc*[tw] OR targeted sequenc*[tw] OR genetic screen*[tw] OR genetic predisposition test*[tw] OR genetic predictive test*[tw] OR genetic test*[tw] OR Genotyping Technique*[tw] OR sequence analys*[tw] OR sequence determination*[tw] OR Quantitative trait loci[tw] OR Quantitative trait locus[tw] |
| #2 | (Scoliosis[Mesh] OR Scolioses[tw] OR Scoliosis[tw]) AND idiopathic[tw] |
| #3 | #1 AND #2 |
| #4 | English[Language] |
| #5 | #3 AND #4 |

| Embase (via Elsevier, Embase.com, 1947 to present) | |
| --- | --- |
| Search date = 04/16/2021 | |
| #1 | Whole Genome Sequencing'/exp |
| #2 | 'genetic association study'/exp |
| #3 | 'copy number variation'/exp |
| #4 | 'sequence analysis'/exp |
| #5 | 'genetic screening'/exp |
| #6 | genotyping technique'/exp |
| #7 | single nucleotide polymorphism'/exp |
| #8 | 'genetic predisposition'/exp |
| #9 | (genetic NEAR/2 (stud* OR variant*)):de,ab,ti |
| #10 | ((DNA or RNA or "next generation") NEAR/3 sequenc*):de,ab,ti |
| #11 | copy number variat*':de,lnk,ab,ti |
| #12 | (LBX1 OR GPR126 OR TBX6):de,lnk,ab,ti |
| #13 | (("Single Nucleotide" or "Copy Number") NEAR/3 Polymorphism*):de,lnk,ab,ti |
| #14 | copy number change*':de,lnk,ab,ti |
| #15 | (Genetic NEAR/2 Association NEAR/2 stud*):de,lnk,ab,ti |
| #16 | ('Genotype Phenotype' NEAR/3 (Association* OR Correlation*)):de,lnk,ab,ti |
| #17 | ("Candidate Gene" NEAR/3 (Identification* or Analys* or stud*)):de,lnk,ab,ti |
| #18 | Gene Discover*':de,lnk,ab,ti |
| #19 | ((Complete or whole) NEAR/1 (Genome or transcriptome or exome) NEAR/1 Sequenc*):de,lnk,ab,ti |
| #20 | (genetic NEAR/3 (screen* OR test*)):de,ab,ti |
| #21 | Genotyping Technique*':de,lnk,ab,ti |
| #22 | (sequence NEAR/3 (analys* or determination* or targeted)):de,lnk,ab,ti |
| #23 | ("Quantitative trait" NEAR/1 (loci OR locus)):de,lnk,ab,ti |
| #24 | #1 OR #2 OR #3 OR #4 OR #5 OR #6 OR #7 OR #8 OR #9 OR #10 OR #11 OR #12 OR #13 OR #14 OR #15 OR #16 OR #17 OR #18 OR #19 OR #20 OR #21 OR #22 OR #23 |
| #25 | idiopathic scoliosis'/exp |
| #26 | (Scolios?s AND idiopathic):de,lnk,ab,ti |
| #27 | #25 OR #26 |
| #28 | #27 AND #24 |
| #29 | English:la |
| #30 | #28 AND #29 |

| MEDLINE (via Ovid MEDLINE® ALL, 1946 to present) | |
| --- | --- |
| Search date = 04/16/2021 | |
| 1 | exp Whole Genome Sequencing/ |
| 2 | exp Genetic Association Studies/ |
| 3 | exp Genome-Wide Association Study/ |
| 4 | exp DNA Copy Number Variations/ |
| 5 | exp Sequence Analysis, DNA/ |
| 6 | exp Sequence Analysis, RNA/ |
| 7 | High-Throughput Nucleotide Sequencing/ |
| 8 | exp Genetic Testing/ |
| 9 | exp Genotyping Techniques/ |
| 10 | exp Polymorphism, Single Nucleotide/ |
| 11 | exp Sequence Analysis/ |
| 12 | exp "Genetic Predisposition to Disease"/ |
| 13 | (genetic ADJ2 (stud* OR variant*)).tw,kf. |
| 14 | ((DNA or RNA or "next generation") adj3 sequenc*).tw,kf. |
| 15 | copy number variat*.mp |
| 16 | (LBX1 OR GPR126 OR TBX6).mp |
| 17 | (("Single Nucleotide" or "Copy Number") ADJ3 Polymorphism*).mp |
| 18 | copy number change*.mp |
| 19 | (Genetic ADJ2 Association ADJ2 stud*).mp |
| 20 | (Genotype-Phenotype ADJ3 (Association* OR Correlation*)).mp |
| 21 | ("Candidate Gene" adj3 (Identification* or Analys* or stud*)).mp |
| 22 | Gene Discover*.mp |
| 23 | ((Complete or whole) adj (Genome or transcriptome or exome) adj Sequenc*).mp |
| 24 | (genetic ADJ3 (screen* OR test*)).tw,kf. |
| 25 | Genotyping Technique*.mp |
| 26 | (sequence adj3 (analys* or determination* or targeted)).mp |
| 27 | ("Quantitative trait" ADJ (loci OR locus)).mp |
| 28 | 1 OR 2 OR 3 OR 4 OR 5 OR 6 OR 7 OR 8 OR 9 OR 10 OR 11 OR 12 OR 13 OR 14 OR 15 OR 16 OR 17 OR 18 OR 19 OR 20 OR 21 OR 22 OR 23 OR 24 OR 25 OR 26 OR 27 |
| 29 | exp Scoliosis/ |
| 30 | Scolios?s.mp |
| 31 | 29 OR 30 |
| 32 | idiopathic.mp |
| 33 | 32 AND 31 |
| 34 | 33 AND 28 |
| 35 | English.lg |
| 36 | 34 AND 35 |
| 37 | Remove duplicates from 36 |

| Web of Science Core Collection (via Clarivate Analytics, including Science Citation Index Expanded and Social Sciences Citation Index, 1974 to present) | |
| --- | --- |
| Search date = 04/16/2021 | |
| #1 | TS=(genetic NEAR/2 (stud* OR variant*)) |
| #2 | TS= ((DNA or RNA or "next generation") NEAR/3 sequenc*) |
| #3 | TS=copy number variat*' |
| #4 | TS=(LBX1 OR GPR126 OR TBX6) |
| #5 | TS=(("Single Nucleotide" or "Copy Number") NEAR/3 Polymorphism*) |
| #6 | TS=copy number change*' |
| #7 | TS=(Genetic NEAR/2 Association NEAR/2 stud*) |
| #8 | TS=("Genotype Phenotype" NEAR/3 (Association* OR Correlation*)) |
| #9 | TS=("Candidate Gene" NEAR/3 (Identification* or Analys* or stud*)) |
| #10 | TS=Gene Discover*' |
| #11 | TS=((Complete or whole) NEAR/1 (Genome or transcriptome or exome) NEAR/1 Sequenc*) |
| #12 | TS=(genetic NEAR/3 (screen* OR test*)) |
| #13 | TS=Genotyping Technique*' |
| #14 | TS=(sequence NEAR/3 (analys* or determination* or targeted)) |
| #15 | TS=("Quantitative trait" ADJ (loci OR locus)) |
| #16 | #1 OR #2 OR #3 OR #4 OR #5 OR #6 OR #7 OR #8 OR #9 OR #10 OR #11 OR #12 OR #13 OR #14 OR #15 |
| #17 | TS=(Scolios?s AND idiopathic) |
| #18 | #16 AND #17 |
| #19 | #18 AND Language:{English} |

| Google Scholar | idiopathic adolescent scoliosis "genetic\|genome\|DNA\|RNA study\|sequencing\|analysis\|association" |
| --- | --- |
