# Supplementary material for: Genetic variants associated with the occurrence and progression of adolescent idiopathic scoliosis: a systematic review protocol
Source: Syst Rev. 2022 Jun 9;11:118. doi: 10.1186/s13643-022-01991-8 (PMC9178937; doi:10.1186/s13643-022-01991-8)
Supplement: Supplementary file 3 — Additional file 3. Inclusion Exclusion [file 13643_2022_1991_MOESM3_ESM.docx]

Supplemental File 3: Inclusion and Exclusion Criteria

| **Inclusion Criteria** | **Exclusion Criteria** |
| --- | --- |
| Adolescent Idiopathic Scoliosis  Idiopathic Scoliosis  Genome Wide Association Study (GWAS)  Next-generation sequencing  Targeted sequencing  Exome sequencing  Whole genome sequencing  Pediatric  Case-control studies  Validation studies | Neuromuscular scoliosis  Infantile scoliosis  Marfan syndrome  Ehlers-Danlos syndrome  Linkage study  Non-idiopathic scoliosis  Degenerative scoliosis  Connective tissue disorder  Syndromic scoliosis  Spondylolysis  Case-only studies  Meta-analyses  Systematic reviews  Review articles  Case numbers <100 subjects |
